# Validation of HepG2/C3A Cell Cultures in Cyclic Olefin Copolymer Based Microfluidic Bioreactors

**DOI:** 10.3390/polym14214478

**Published:** 2022-10-22

**Authors:** Leire Etxeberria, Taha Messelmani, Jon Haitz Badiola, Andreu Llobera, Luis Fernandez, José Luis Vilas-Vilela, Eric Leclerc, Cécile Legallais, Rachid Jellali, Ane Miren Zaldua

**Affiliations:** 1 Leartiker S. Coop., Xemein Etorbidea 12, 48270 Markina-Xemein, Spain; 2 microLIQUID S.L, Goiru 9, 20500 Arrasate-Mondragon, Spain; 3 Macromolecular Chemistry Research Group (labquimac), Department of Physical Chemistry, Faculty of Science and Technology, University of the Basque Country (UPV/EHU), 48940 Leioa, Spain; 4 CNRS, Biomechanics and Bioengineering, Centre de Recherche Royallieu-CS 60319, Université de Technologie de Compiègne, 60203 Compiègne, France; 5 BC Materials, Basque Center for Materials, Applications and Nanostructures, UPV/EHU Science Park, 48940 Leioa, Spain; 6 CNRS IRL 2820, Laboratory for Integrated Micro Mechatronic Systems, Institute of Industrial Science, University of Tokyo, 4-6-1 Komaba, Tokyo 153-8505, Japan

**Keywords:** cyclic olefin copolymer, injection moulding, Organ-on-Chip, liver, cell growth

## Abstract

Organ-on-chip (OoC) technology is one of the most promising in vitro tools to replace the traditional animal experiment-based paradigms of risk assessment. However, the use of OoC in drug discovery and toxicity studies remain still limited by the low capacity for high-throughput production and the incompatibility with standard laboratory equipment. Moreover, polydimethylsiloxanes, the material of choice for OoC, has several drawbacks, particularly the high absorption of drugs and chemicals. In this work, we report the development of a microfluidic device, using a process adapted for mass production, to culture liver cell line in dynamic conditions. The device, made of cyclic olefin copolymers, was manufactured by injection moulding and integrates Luer lock connectors compatible with standard medical and laboratory instruments. Then, the COC device was used for culturing HepG2/C3a cells. The functionality and behaviour of cultures were assessed by albumin secretion, cell proliferation, viability and actin cytoskeleton development. The cells in COC device proliferated well and remained functional for 9 days of culture. Furthermore, HepG2/C3a cells in the COC biochips showed similar behaviour to cells in PDMS biochips. The present study provides a proof-of-concept for the use of COC biochip in liver cells culture and illustrate their potential to develop OoC.

## 1. Introduction

The liver performs a great number of tasks to support other organs and impacts all physiological system. It is involved in several essential functions including detoxification, protein synthesis and metabolism, glycogen storage, bile secretion, as well as the removal of pathogens and exogenous antigens form the systemic circulation [1,2]. As the major site of xenobiotic metabolism, the liver is exposed to a wide range of exogenous substances and is often the subject of chemical-induced toxicity [2]. Therefore, the investigation of drugs/chemicals hepatotoxicity is critical to their successful use.

Most of the standard toxicological approaches for evaluating chemical toxicity involve complex in vivo animal studies, which are both time consuming and costly. Moreover, the data provided by animal models cannot always be extrapolated to human metabolic situation due to the complexity of the metabolism reactions in the human body [3,4,5]. Due to the concern of animal welfare and cost of the experiments, in vitro culture systems become highly interesting for toxicity studies. However, the conventional in vitro methods are mainly based on static 2D cultures in Petri dishes, which are poorly representative of human in vivo physiology, metabolism and toxicity [6].

Among the new technologies, Organ-on-a-Chip (OoC) devices based in microfluidics are extremely attractive for in vitro pharmaceutical and toxicological studies. The progress made in microtechnology allows to improve in vitro cultures and construct relevant in vitro models with complex geometries [7,8,9,10,11,12]. The dynamic OoC cultures allows to reproduce several characteristics of in vivo environment such as physiological shear stress, three-dimensional organization, dynamic flow, zonation and homogenous transport of molecules such as hormones, drugs and metabolic waste [13]. Thus, several advanced works for bioartifical in vitro Liver-on-a-Chip (LoC) devices has been published in the last years [14,15,16,17,18,19].

One of the main functions of a LoC devices is to control the growth and the cell behaviour in the microenvironment, so the selected material for the manufacturing on the device is crucial for the correct development of the analysis [20]. Polymer based microfluidic devices are of great interest due to their light weight, low cost, optical transparency and chemical resistance [21,22]. Due to its properties and the facility of prototyping manufacturing, polydimethylsiloxane (PDMS) is the most used material for microfluidic devices due to its biocompatibility and ease of transformation [23,24,25]. However, different studies showed several disadvantages associated with the use of the PDMS in microfluidic devices as water absorption, non-specific adsorption of molecules and porosity among others [26,27]. To date, thermoplastic polymers, such as Polycarbonate (PC), Polymethyl-methacrylate (PMMA), Polyestyrene (PS) and Cyclic Olefin Polymer (COP), in particular Cyclic Olefin Copolymers (COC), have emerged as the most promising materials for the mass manufacturing of low-cost microfluidic devices [28,29].

COCs are engineering thermoplastics produced by copolymerization of cyclic monomers like norbornene with ethylene. It has a fully carbon-based main chain and no double bonds as shown in Figure S1 (Supplementary File). COCs show a unique combination of properties such as glass-like optical transparency, low water absorption, excellent water-vapour barrier properties, low dielectric loss, high heat resistance, biocompatibility and low-cost. In terms of chemical properties, COCs show resistance to inorganic acids and bases as well as polar organic solvents [30,31]. Furthermore, scaling up the production of organ-on-a-chip devices is one important issues to overcome as most devices analysed in literature is based on laboratory based materials, such as PDMS, and fabrication methods [32,33]. Injection moulding of thermoplastics, such as COC, is reported to be cheap, fast and promising approach for a massive fabrication of Organ-on-chip devices [34,35,36].

In this work, the manufacturing of a COC biochip was performed after adapting it for mass production by injection moulding. The biochip is based on previous design, that showed good performances as LoC manufactured with PDMS and Perfluoropolyethers, being both of them biocompatible [37,38,39,40,41,42]. The validation of the COC biochip for a Liver-on-a-chip bioreactor was performed. The growth, viability, behaviour and metabolism of HepG2/C3A cells, which are commonly used as liver cell model, were studied and compared with results obtained using PDMS biochips.

## 2. Materials and Methods

### 2.1. Materials

The selected materials have been polydimethylsiloxane (PDMS) and Cyclic Olefin Copolymer (COC). The used PDMS was Sylgard 184 kit (PDMS polymer + curing agent; 10:1 mixing ratio) purchased from Dow Corning (Midland, TX, USA). COC polymer was purchased from TOPAS Advanced Polymers (Raunheim, Germany).

### 2.2. Design and Manufacturing of the Bioreactor

**Figure 1:**
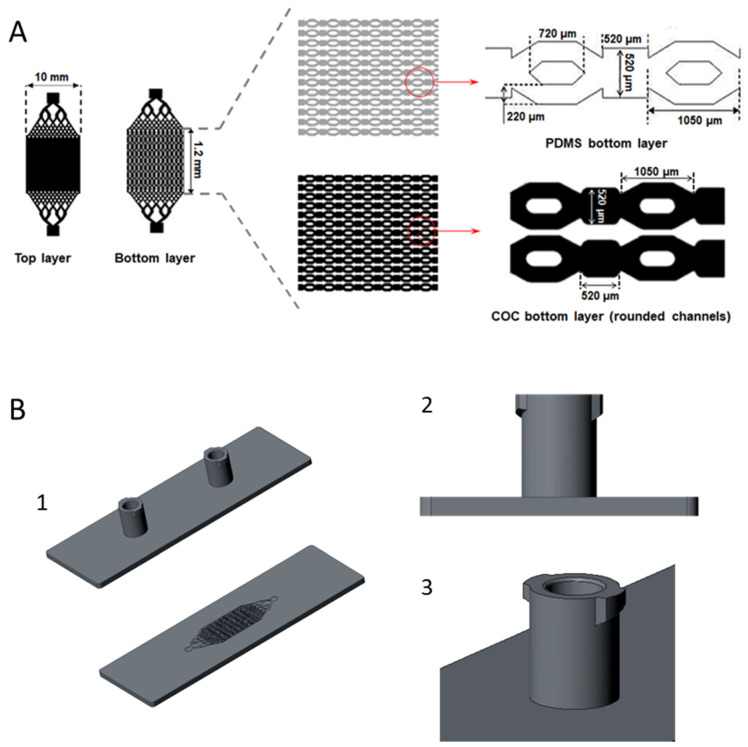
(**A**) Design of the biochip layers, (**B**) CAD design of COC bioreactor: (1) complete Biochip, (2) and (3) Luer lock connectors.

The biochip consists of 2 parts: the microstructured bottom layer contains cell culture chambers and microchannels (high of 100 µm), and the top layer, with a reservoir 100 µm in depth, includes an inlet and outlet for culture medium perfusion. The aim of the design is to allow a uniform flow within the microstructures. The microstructure design and fabrication of the bioreactor has been reported in previous works (Figure 1A) [37,38,39,40]. The PDMS layers was manufactured by replica moulding process using SU-8 (epoxy-based negative photoresist) mould fabricated by photolithography process. Then, the two layers were sealed together after surfaces activation with reactive air plasma (1 min; Harrick Scientific, Pleasantville, NY, USA).

Being the aim of this work to validate COC for the mass manufacturing of LoC devices, the design of the microstructures and microchambers has been updated to adapted to mass manufacturing processes. Rounded etches, typically obtained after a machining process of injection moulding tools, have been introduced in the biochips. The modified geometries of the bottom layer for COC devices are shown in Figure 1B. In the case of the biochip top layer, the adaptation for scaling purpose has been performed adding Luer lock connectors, one of the gold standards for medical and laboratory instruments, such as needles, syringes, and cannulas due to its easy handling and compatibility [43,44]. The design of the cover containing Luer lock connectors based on ISO 80369-7 [45] are shown in Figure 1B.

The injection moulding process has been carried out in Arburg 270 injection machine. The injection mould was designed using the software CREO Parametrics (6.0.5.1) and the machining and manufacturing of the inserts and mould was carried out in Ruimoldes 2012 S.L. The clamping force of the machine is 25 T, screw of 20 mm diameter and 80 cm^3^ of maximum injection volume. The selected injection parameters were; a melt temperature of 270 °C, mould temperature of 30 °C and injection speed of 13 mm/s. The bonding of the biochip was performed using a doble-sided pressure sensitive adhesive.

### 2.3. Scanning Electron Microscopy

The PDMS and COC microstructured layers were characterized by scanning electron microscopy (SEM) using a Quanta 250 FEG microscope (Thermofischer, Eindhoven, The Netherland). The samples were recovered by a thin palladium layer prior to analysis and the images were acquired with a 20 kV accelerating voltage using the hivac mode.

### 2.4. Flow and Pressure Measurements

Flow and pressure measurements have been performed to analyse the hydraulic resistance of the biochips. The fluidic circuit for the analysis consists of a pressure controller (MFCS-EX, Fluigent) that was connected the biochip and to a flow sensor (Flow Unit type M, Fluigent). PEEK tubes connected all the circuit to allow the culture medium to flow into the biochip. A feedback loop adjusted the pressure applied in the reservoir to ensure the maintenance of the desired flow rate. The schematic representation of the microfluidic circuit is shown in Figure S2 (Supplementary File).

The pressure differential is generated by pressurizing the inlet and the outlet, and the resultant flow rate is measured for each biochip. The hydraulic resistance of the circuit is calculated following the Equation (1):

(1) $$R_{H}=\frac{\Delta P}{Q}$$

where ΔP is the pressure differential between inlet and outlet and Q is the flow rate. The hydraulic resistance of PEEK tubing within the circuit is determined by Equation (2):

(2) $$r_{h}=\frac{8\cdot \mu \cdot l}{\pi \cdot R^{4}}$$

where μ is the viscosity of the fluid, *l* is the length of the tube, and *R* is the internal radio of tube. The hydraulic resistance of the biochips is calculated by subtracting the hydraulic resistance of PEEK tubes to the hydraulic resistance of the whole circuit.

### 2.5. Cells

HepG2/C3a hepatocarcinoma cells were provided by the American Type Culture Collection (ATCC, CRL-10741). The used culture media contains Minimal Essential Medium (MEM, Gibco, Waltham, MA, USA), 2 mM L-Glutamine (Gibco), 0.1 mM non-essential amino acids (Gibco), 1 mM sodium pyruvate (Gibco), 10% (*v*/*v*) fetal bovine serum (Gibco) and penicillin-streptomycin (100 units/mL–100 µg/mL, Pan Biotech, Aidenbach, Germany). The batch cultures were performed in T75 flasks (Falcon, Merk Eurolab, Strasbourg, France) using 15 mL of culture medium and the cells were maintained at 37 °C in a humidified atmosphere supplied with 5% of CO_2_. The cells were passaged weekly at a confluence of 80–90% and the culture medium was renewed every two days.

### 2.6. Dynamic Culture in Biochip

**Figure 2:**
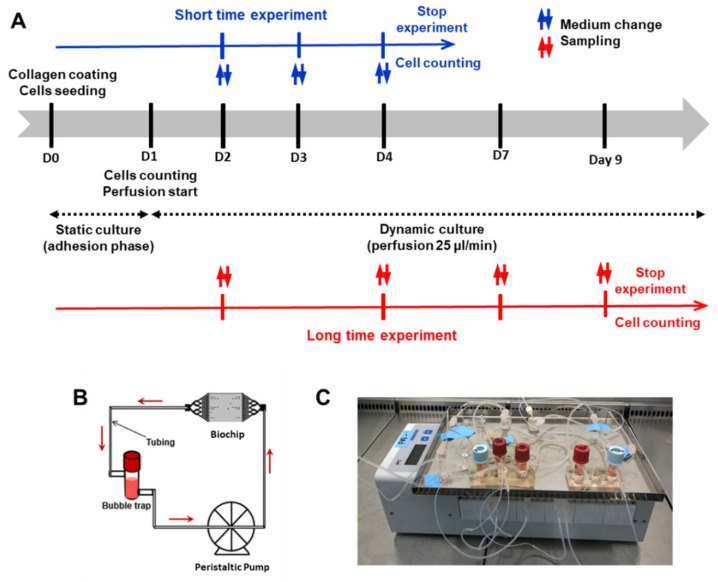
Design of the experiments: (**A**) Timing and steps of short and long-time experiments including the adhesion and perfusion phases, (**B**,**C**) schematic representation ad image of dynamic flow setup respectively.

Two dynamic culture experiments of different durations have been performed, both including two phases: the adhesion phase (24 h) and the perfusion phase (3 and 8 days). Figure 2A shows the timing and steps of both short and long-time experiments. The experimental setup used for dynamic cells culture was composed of a perfusion loop, including the culture medium tank (bubble trap), the peristaltic pump and one biochip. They were interconnected using 0.65 mm interior diameter silicone/Teflon tubing (Figure 2B). Before each experiment, the tubing and bubble trap were sterilized by autoclaving, while the biochips (PDMS and COC) were sterilized using ethanol (70%).

To enhance the cell adhesion, before the seeding of the cells, the bioreactors were coated with rat tail type 1 collagen (Corning, NY, USA; 300 µg/mL in buffer saline solution: PBS Gibco) and incubated at 37 °C in an atmosphere supplied with 5% CO_2_. After 1 h, the cleaning of the collagen was performed with culture medium and 0.2 ± 0.03 × 10^5^ cells were inoculated inside of each biochip. The cells were incubated in static conditions for adhesion during 24 h in a 5% CO_2_ incubator at 37 °C. To keep the culture medium inside the culture chamber, the biochip inlet ports were closed using two syringes (containing 500 μL of culture medium).

After 24 h of adhesion, 3 biochips were chosen for initial cell counting (adherent cell) and the rest were prepared for the perfusion phase. The biochips were then connected to the perfusion loop, and 2 mL of culture medium were added in each bubble trap (Figure 2A,B). The entire setup was incubated at 37 °C in a 5% CO_2_ supplied incubator and the peristaltic pump was started at flow rate of 25 µL/min. The medium was collected (for subsequent analysis) and renewed every day.

### 2.7. Cell Counting and Viability

Cell counting was made by detachment with trypsin-EDTA (0.25%, Gibco) and counting using a graduated Malassez cells. The proliferation rate was calculated by dividing the cell number counted at the end by the number of seeded cells. Trypan blue staining was used for viability analysis.

Each analysis was repeated three times in triplicate for each material and three biochips were counted for each experiment (3 experiments × 3 biochips = 9 replicates). Data is plotted as mean ± SD.

### 2.8. Albumin Measurements

The albumin produced by cells and released in culture medium measurements were measured using ELISA sandwich test in a 96-well plate. The assays were performed using a human albumin ELISA Quantitation Set (E80-129, Bethyl Laboratories, Montgomery, TX, USA), following the manufacturer instructions. The plate was read with an absorbance wavelength of 490 nm, using a Spectafluor Plus microplate reader (TECAN, Männedorf, Switzerland). The analyses were performed with culture medium collected from three biochips and repeated three time in replicate, leading to *n* = 9 (3 experiments × 3 biochips = 9 replicates). Data is plotted as mean ± SD.

### 2.9. Immunostaining and Confocal Microscopy

After adhesion and perfusion phases (end of the experiment), the biochips were washed with PBS (Gibco), fixed in paraformaldehyde 4% (PFA, MP biomedicals, Illkirch-Graffenstaden, France) for 30 min at room temperature and washed and stored in PBS until staining. Cell nuclei were stained with DAPI at 10 µg/mL (4′,6-diamidino-2-phenylindole, D1306, Invitrogen) and phalloidin (Alexa Fluo 488 Phalloidin, Thermo Fisher (Waltham, MA, USA)) staining was used for F-actin visualization. The samples were incubated, in dark and at room temperature, with phalloidin for 3 h and DAPI for 30 min. At the end of the incubation, the samples were washed with PBS.

The observations of the stained samples were made with a laser scanning confocal microscope (SM 710, Zeiss (Oberkochen, Alemania)) at 647 and 488 nm, respectively.

## 3. Results

### 3.1. Biochips Characterization

**Figure 3:**
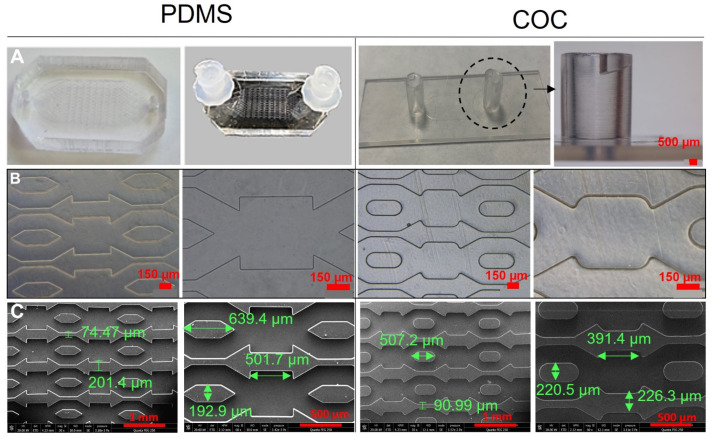
COC and PDMS biochips characterization: (**A**) biochips pictures, (**B**,**C**) phase contrast microscopy and MEB images, respectively.

The pictures of COC (injection moulding) and PDMS (replica moulding) biochips are presented in Figure 3A. The injection moulding of COC allows to produce very thin biochip with good optical transparency. The design of the biochip facilitates the connection with standard Luer lock connectors (typically used for medical devices), and thus, the world-to-chip interconnection for cell inoculation and dynamic cell culture. In the case of PDMS biochips, the used Luer lock connectors are commercial and are added to the biochips after the manufacturing of the complete biochips whereas in the case of COC biochips, and due to the adaptation of the design to mass manufacturing purposes, Luer lock connectors are part of the injection moulded cover as can be observed in Figure 3A.

Figure 3B shows the phase contrast microscopy observations of the bottom layer of the biochips manufactured with PDMS and COC materials. The flexibility of the PDMS makes the demoulding easier for PDMS than for COC. Nevertheless, the replicas obtained in COC are precise and maintain structures dimensions. The microstructured bottom layers (PDMS and COC) were also analysed using SEM. The images are presented in Figure 3C. They show an accurate replication of the microstructures in both materials, good accuracy in the dimensions, excellent surface qualities and no deformations in the microstructures. This confirms an optimum moulding condition regardless the material (COC or PDMS) and technology used (injection moulding or replica moulding). 

### 3.2. Flow and Pressure Measurements

To evaluate the biochips sealing and the flow circulation inside the bioreactors, we performed a dynamic test in which several flow rates and pressure were tested (experimental detail in Section 2.4). Regarding the maximum outlet pressure that the biochips can withstand, COC biochips started leaking at 400 mbar whereas PDMS biochips can withstand 500 mbar outlet pressures. Nevertheless, for COC biochip, no leakage was observed in continuous perfusion at a pressure of 300 mbar.

**Figure 4:**
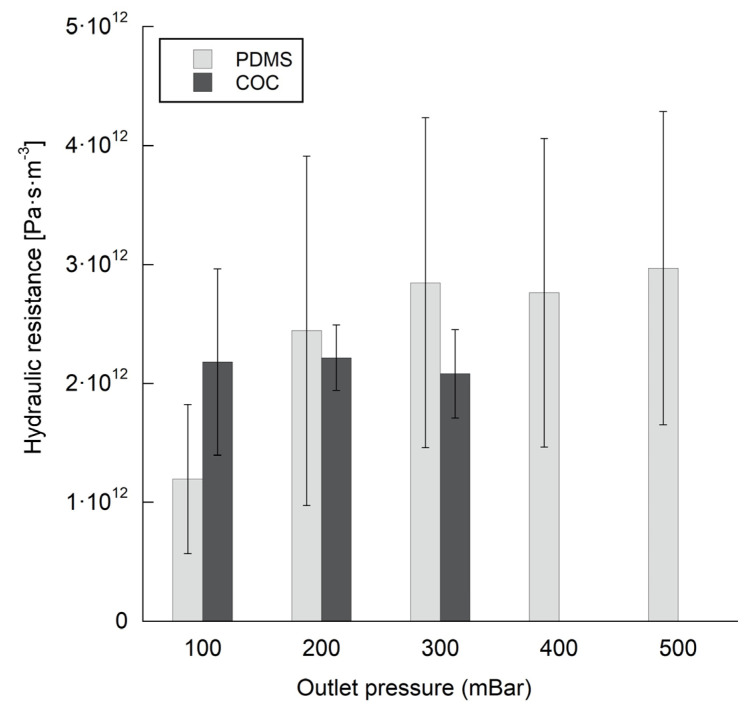
Hydraulic resistance for PDMS and COC Biochips.

Using the equations 1 and 2 (see Section 2.4), we calculated the hydraulic resistance of COC and PDMS biochips. The results obtained during the flow and pressure measurements are presented in Figure 4. No significant difference was observed between the two types of biochips. The hydraulic resistances were approximately of 2.2·10^12^ ± 0.35·10^12^ and 2.75·10^12^ ± 1.3·10^12^ Pa·s·m^−3^ for COC and PDMS biochips, respectively. The hydraulic resistance values of COC biochips show less standard variation than PDMS biochipswhich can be due to the manual manufacturing steps typically used for PDMS biochip fabrication.

### 3.3. HepG2/C3a Cell Adhesion on COC and PDMS Biochips

**Figure 5:**
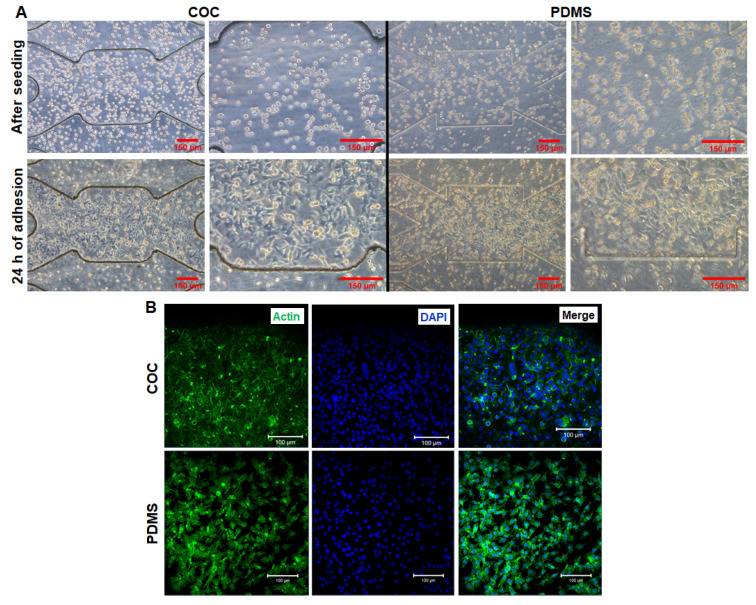
Characterization of HepG2/C3a cells adhesion on COC and PDMS biochips: (**A**) cell morphology after seeding and after 24 h of adhesion (scale bar 150 µm), (**B**) Phalloidin and DAPI stainings of cells after adhesion phase: DAPI (nuclei, blue) and phalloidin (F-actin, green); scale bar 100 µm.

Cells adhesion on the bottom microstructured layer of biochip is crucial before starting the dynamic perfusion. For this, a first serie of experiments was performed in order to investigate the behaviour of HepG2/C3a cells in contact with COC substrate. The COC and PDMS (control) biochips were preliminarily coated with collagen. The cells morphology after seeding are presented in Figure 5A. In both COC and PDMS biochips, the cells present rounded shape and were in suspension in the culture chamber. Furthemore, the HepG2/C3a cells were homogeneously dispersed throughout the microchambers and microchannels of the bioreactors.

After 24 h of adhesion phase in static conditions, the culture medium was changed to remove non-adherent cells and the biochips were observed using phase contrast microscopy. Figure 5A presents the cell morphology inside the biochips. The adhesion of HepG2/C3a to the bottom surfaces of the microfluidic bioreactors was successful for both materials used (COC and PDMS). We did not detect any significant difference between COC and PDMS. The cells exhibited an elongated shape and homogeneously occupy the surface of the culture chambers. The cell counting performed post-adhesion (after cell detachment with trypsin) revealed 0.185 ± 0.02 × 10^5^ and 0.15 ± 0.007 × 10^5^ attached cells in COC and PDMS biochips, respectively. In comparison with the initial seeded cells, the percentage of adhered cells was of 90 ± 5% for COC biochip and 75 ± 8% for PDMS biochip. Finally, the cytoskeleton organization was investigated by actin staining. As shown in Figure 5B and Figure S3 (Supplementary File), the actin cytoskeleton of the cells can be seen clearly (intense green fluorescence signal) in the whole culture chamber. The actin filaments appear to be concentrated beneath the cell membrane (around nuclei).

### 3.4. Short Time Dynamic Culture of HepG2/C3a in Biochips

After adhesion validation and to evaluate the potential of COC biochips for dynamic cell culture, we performed a series of dynamic cultures for short time. These cultures were performed for 4 days: 24 h of adhesion and 72 h of dynamic perfusion. For comparison, the experiments were realized with COC and PDMS biochips

**Figure 6:**
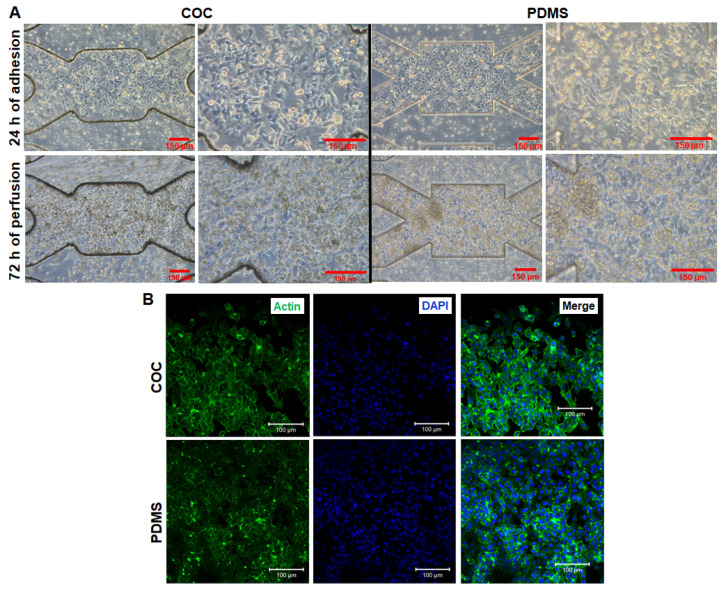
Characterization of dynamic cultures in biochip: (**A**) cell morphology after adhesion and at the end of 72 h of perfusion (scale bar 150 µm), (**B**) Phalloidin and DAPI stainings of cells at the end of perfusion: DAPI (nuclei, blue) and phalloidin (F-actin, green); scale bar 100 µm.

**Figure 7:**
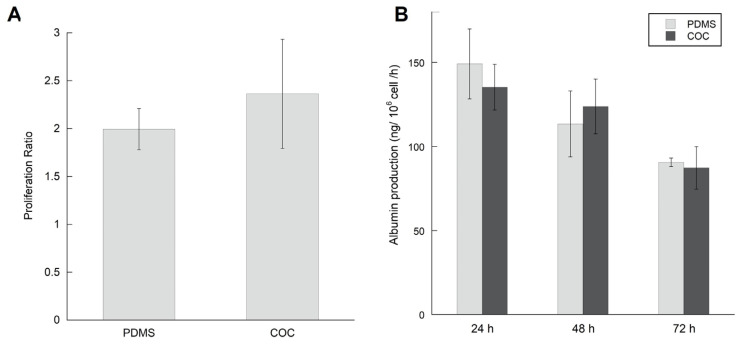
Proliferation and functionality of HepG2/C3a cells cultured in dynamic COC and PDMS biochips for 96 h including 24 h of adhesion and 72 h of perfusion: (**A**) Proliferation Ratio at the end of the experiment, (**B**) Albumin production after 24 h, 48 h and 72 h of perfusion.

The morphology of HepG2/C3a cells after adhesion phase and at the end of the experiment (24 h of adhesion + 72 h of dynamic culture) is presented in Figure 6A. As observed in the previous Section 3.3, the cells have successfully adhered to the biochips. The cell proliferation was evident between the first 24 h of culture (after adhesion) and the end of the experiment (72 h of perfusion). The cells have proliferated over confluence and started to form a second layer. As cell density was high, it is difficult to distinguish the cellular phenotype. We were not able to observe a difference between the morphologies of cells cultured in COC biochips and cells cultured in PDMS biochips. The cell detachment at the end of the experiments allowed cells counting and viability assessment (trypan blue staining). The proliferation ratio was of 2.3 ± 0.6 and 2 ± 0.3 for COC and PDMS biochips, respectively (Figure 7A). Moreover, the viability tests performed by trypan blue after 96 h of culture (24 h static culture and 72 h of perfusion) revealed good viability in both cultures’ conditions (viability above 90%). The phalloidin staining confirmed the development of actin cytoskeleton in COC and PDMS culture (no specific difference was detected between the two materials, Figure 7B and Figure S3, Supplementary File).

To evaluate the functionality of HepG2/C3a cells cultured in COC biochips, albumin production was monitored throughout the 72 h of perfusion and compared with the results obtained in PDMS biochips. The results are shown in Figure 7B. the albumin production of cells cultured in COC bioreactors was of 135 ± 27, 123 ± 32 and 87 ± 25 ng/h/10^6^ cells after 24, 48 and 72 h of perfusion, respectively. The quantity of albumin produced by HepG2/C3a cells in PDMS biochips was similar: 149 ± 41 (24 h), 113 ± 39 (48 h) and 90 ± 5 (72 h) ng/h/10^6^ cells.

### 3.5. HepG2/C3a Cells Behaviour in Long Time Experiment

**Figure 8:**
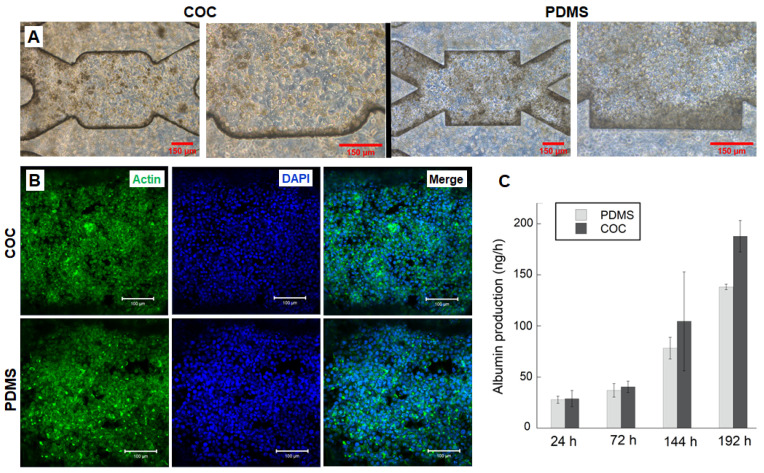
Characterization of HepG2/C3a cells after 9 days of culture in COC and PDMS biochips: (**A**) cell morphology (scale bar 150, (**B**) Phalloidin and DAPI stainings: DAPI (nuclei, blue) and phalloidin (F-actin, green); scale bar 100 µm, (**C**) Albumin production throughout the 8 days of perfusion.

The HepG2/C3a cultures in COC biochips were also performed for long time and compared to cultures in PDMS biochips. The duration of the cultures was of 9 days, including 24 h of adhesion and 8 days of dynamics perfusion at 25 µL/min. The morphology of the cells at the end of the experiments are presented in Figure 8A. In both COC and PDMS biochips, the cells proliferated in multilayer leading to the formation of a dense 3D tissue-like structure. Consequently, the cell shapes are difficult to observe. This result was confirmed by actin cytoskeleton staining that showed a dense green staining in the whole culture chambers (Figure 8B and Figure S3, Supplementary File). The z-stack analysis (performed by the confocal microscope) of actin localization confirmed the cell organization in several layers.

The albumin production by HepG2/C3a cells cultured in dynamic COC biochips was quantified over the 9 days of culture and compared to albumin secreted in PDMS biochips. The measured albumin was similar for both biochips. The production gradually increased throughout the 9 days of the experiment (1 day of adhesion + 8 days of perfusion). The productions after 24 h of perfusion were of 29 ± 9 and 27.79 ng/h for COC and PDMS biochips, respectively. At day 8, the albumin productions reached 187 ± 15 ng/h for COC and 140 ± 3 ng/h for PDMS.

## 4. Discussion

In the last decades, multiple approaches based on tissue engineering and microtechnology have been used to develop relevant in vitro models for animal experiments replacement. Thanks to the progress in microfabrication, microfluidic and bioprinting, OoC technology has emerged as a promising tool to recapitulate in vitro the physiological in vivo environment of the cells or tissues [2,20]. Since the beginnings of microfluidics, PDMS has been the most commonly used material for microfluidics and OoC devices and is still today a widely used material in literature [46,47,48]. The choice of PDMS was motived by its several advantages such as biocompatibility, low cost, optical transparency and gas permeability [49]. However, PDMS is not suitable for drug screening and toxicity studies due to its strong absorption of molecules [27,50]. Furthermore, PDMS casting is a time-consuming process difficult to scale up the production.

Thus, devices made of non-absorbent material are required to improve the use of OoC platforms for chemicals testing. Among other materials, COC has properties suitable for use in drug discovery. In addition to its transparency and biocompatibility, COC exhibits no/low absorption of chemicals and is approved by Food and Drug Administration (FDA) [51,52]. Despite these interesting properties, the COC devices are not widely used in cell culture [53,54,55]. The current work aims to develop standardized COC bioreactor using process adapted to mass production and to validate its use for cell culture.

The COC bioreactors were manufactured by injection moulding using design previously used for PDMS biochips fabrication and studied with several cells [18,37,38,56]. As COC is more rigid than PDMS, the design of the biochip bottom layer (microstructured layer) was adapted to injection moulding (to obtain better replication and easy demoulding). The corners of microchambers and microchannels were rounded. The COC biochips obtained using the modified design showed good accuracy of microfluidic structures and demonstrated that microfluidic small and complex structures can be replicated by injection moulding. The surface of the moulded COC was also homogenous, without any irregularities or artifacts of incomplete replication. These results agree with previous studies in literature reporting easy and good replication of microstructure in COC using injection moulding or hot embossing [55,57,58]. In the redesigning, a standard Luer connectors were added, and the external dimensions were updated to allow mass manufacturing processes. Including the connectors in the bioreactor has facilitate the job of plug/unplug microfluidic tubing for dynamic cultures. Furthermore, the integration of Luer connectors makes the bioreactor compatible with standard connecters used in medical and laboratory instruments and addresses one of the major challenges of the OoC technology, namely the equipment standardization [8,59].

The characterization of fluid circulation inside the COC and PDMS biochips showed similar hydraulic resistance values. This indicates that the new design and the use of COC (in replacement of PDMS) don’t affect the flow parameters. Indeed, the changes in the design are minor and only concern the corners of the microchambers and microchannels. Likewise, COC material exhibits a wettability close to PDMS, with a contact angle between 85 and 90° [60] (PDMS contact angle is around 100° [61]). The sealed COC biochips were able to withstand a pressure of 300 mbar without any leakage. This pressure is higher than the pressure inside the biochip during cell culture at 25 µL/min (10–20 mbar).

The feasibility of cell culture in the COC biochips was investigated using HepG2/C3a cells. The choice of cell sources is critical for the success of in vitro liver models. Primary human hepatocytes (PHHs) are considered the gold standard to build in vitro liver models for studies of drug toxicity and metabolism [49]. However, PHHs present several disadvantages including high variability, rapid de-differentiation and high cost [62]. The use of HepG2/C3A cells line offer a good compromise for the development of the model and its comparison with literature data. Indeed, the HepG2/C3A cells, which express several hepatic markers, are stable, reproducible and low-cost cells source [19]. As COC is a hydrophobic material, it is not favourable to the adhesion of cells, which preferentially adhere to hydrophilic surfaces (contact angle between 50 and 80°) [61]. In this work, collagen type 1 was used to coat both COC and PDMS biochips. Collagen type 1, the most common type of protein, was previously reported for COC and PDMS coating [40,49,53]. In both substrates, 24 h after seeding, cells showed elongated shape and they were homogeneously distributed on culture chambers, being the percentage of adherent cells 90 ± 5% for COC and 75 ± 8% for PDMS. Moreover, the cell morphology and behaviour were similar to those reported in previous works regarding HepG2/C3A cells [38,40].

The Dynamic cultures were performed in two series varying the dynamic perfusion experiment time. In the short time experiment, the dynamic perfusion was applied for 72 h. Proliferation of cells was evident as a second layer of cells was started to form, making difficult to distinguish the cell phenotype. The proliferation ratio and cell viability were similar in COC and PDMS biochip, and close to value reported in previous works with PDMS biochips [38,40]. Regarding albumin throughout the 72 h of experiment, both biochips cultures maintained similar and relatively stable albumin levels, which were also in the range of levels reported in previous studies [40,63]. The maintain of liver in vitro model for long time of culture is essential in drugs discovery. This allows to test chronic exposures to drugs and elucidate the effect of molecules and metabolites accumulation. The HepG2/C3A cells cultured in COC biochips were successfully maintained for 9 days (24 h of static adhesion and 8 days of perfusion). They proliferated generating a multilayer 3D tissue-like structure and the albumin secretion was gradually increased throughout the 8 days of perfusion (proportionally to cell proliferation). The similar behaviour to PDMS cultures highlighted the potential of COC biochips for long term cultures.

Several studies have been published in recent years for Liver-on-a-chip applications underlining the great potential showed to replace animal experiments for drug testing and liver disease modelling although there are still some existing challenges to address [64,65,66]. The design of the biochip described demonstrated their potential for the cultures of different hepatic cells including HepG2/C3A, primary rat and human hepatocytes and induced pluripotent stem cells (hiPSCs) in PDMS material [18,38,56,67]. Being the results obtained with COC biochips similar of them obtained with PDMS, COC biochips could be considered as a promising alternative for non-absorbent liver-on-a-chip models. Culture of primary hepatocytes, hiPSCs and non-parenchymal liver cells, as well as drugs testing needs to be performed to confirm the potential of the COC device. The major drawback of COC biochips is the impermeability to oxygen that can affect cells, especially in long cultures. It has been demonstrated in this work that 8 days long dynamic cultures can be performed with COC biochips. Normoxic levels for liver tissue are reported to be between 10 and 13 %, which can be maintained with oxygen perfusion methods, the addition of oxygen generating chemicals to the perfused fluid or even using hybrid microfluidic devices (combining oxygen-permeable and -impermeable materials) in case longer experiments required higher oxygen consumption [68,69].

## 5. Conclusions

In this study, we described the development of a cyclic olefin copolymer microfluidic device using injection moulding processing. The chosen material and process offer an interesting combination of advantages for OoC application, including biocompatibility, transparency, low absorption of chemicals, low cost and adaptability to mass manufacturing. The injection moulding process allowed the integration of Luer lock connectors in the biochip top layer, facilitating the biochip connection to standard equipment. The cultures of liver HepG2/C3a cells were performed for short and long time to evaluate the dynamic cell culture in the developed COC biochip. The cell proliferation, viability and albumin synthesis showed that the HepG2/C3a cells cultured in the COC biochip remained functional and metabolically active throughout the time of culture (4 and 9 days). The COC cultures exhibited also similar behaviour to cultures in PDMS biochips. This feasibility study showed the potential of COC devices for application as OoC models. We believe that this device is a promising tool and can be applied with other cell types for drug discovery and toxicity studies. The COC properties can overcome the drawbacks of PDMS by limiting chemicals absorption and paving the path for mass production of robust and standardized devices.

## Data Availability

Not applicable.

## Supplementary Materials

The following supporting information can be downloaded at: https://www.mdpi.com/article/10.3390/polym14214478/s1, Figure S1: Chemical structure of Cyclic Olefin Copolymers; Figure S2: Schematics of the setup used for flow and pressure measurements; Figure S3: Phalloidin and DAPI staining’s of HepG2/C3a cells at different steps of experimen: DAPI (nuclei, blue) and phalloidin (F-actin, green); scale bar 200 µm.
