# Supplementary material for: Validation of HepG2/C3A Cell Cultures in Cyclic Olefin Copolymer Based Microfluidic Bioreactors
Source: Polymers (Basel). 2022 Oct 22;14(21):4478. doi: 10.3390/polym14214478 (PMC9655789; doi:10.3390/polym14214478)
Supplement: Supplementary file 1 [file polymers-14-04478-s001.zip › polymers-1964768-supplementary.pdf]

polymers

MDPI

Article

Validation of HepG2/C3A cell cultures in Cyclic Olefin Copolymer based microfluidic bioreactors

Leire Etxeberria [1,2,3,*], Taha Messelmani [4], Jon Haitz Badiola [1], Andreu Llobera [2], Luis Fernandez [2], José Luis Vilas-Vilela [3,5], Eric Leclerc [4,6], Cecile Legallais [4], Rachid Jelalli [4,*,†], Ane Miren Zaldua [1,†]

[1] Leartiker S. Coop., Xemein etorbidea 12, 48270 Markina-Xemein, Spain
[2] microLIQUID S.L, Goiru 9, 20500 Arrasate-Mondragon, Spain
[3] Macromolecular Chemistry Research Group (labquimac), Department of Physical Chemistry, Faculty of Science and Technology, University of the Basque Country (UPV/EHU), Leioa, Spain
[4] Université de technologie de Compiègne, CNRS, Biomechanics and Bioengineering, Centre de recherche Royallieu-CS 60319 -60203 Compiègne Cedex, France
[5] BC Materials, Basque Center for Materials, Applications and Nanostructures, UPV/EHU Science Park, Leioa, Spain
[6] CNRS IRL 2820, Laboratory for Integrated Micro Mechatronic Systems, Institute of Industrial Science, University of Tokyo, 4-6-1 Komaba, Meguro-ku, Tokyo, Japan
* Correspondence: letxeberria@leartiker.com (L.E); rachid.jellali@utc.fr (R.J)
† These authors contributed equally to this paper.

Supplementary figures

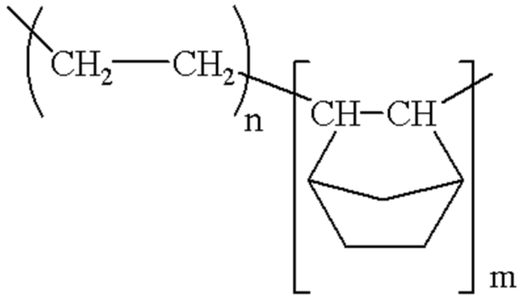

Figure S1. Chemical structure of Cyclic Olefin Copolymers.

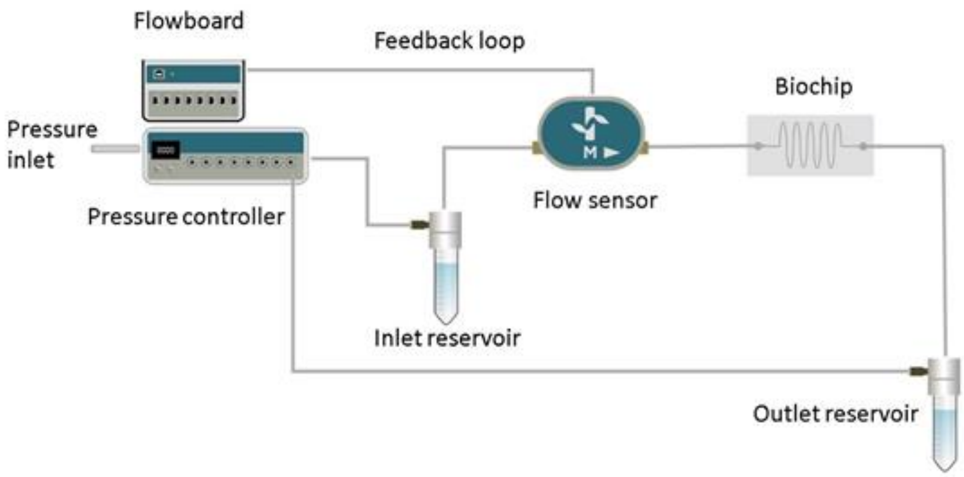

Figure S2. Schematics of the setup used for flow and pressure measurements.

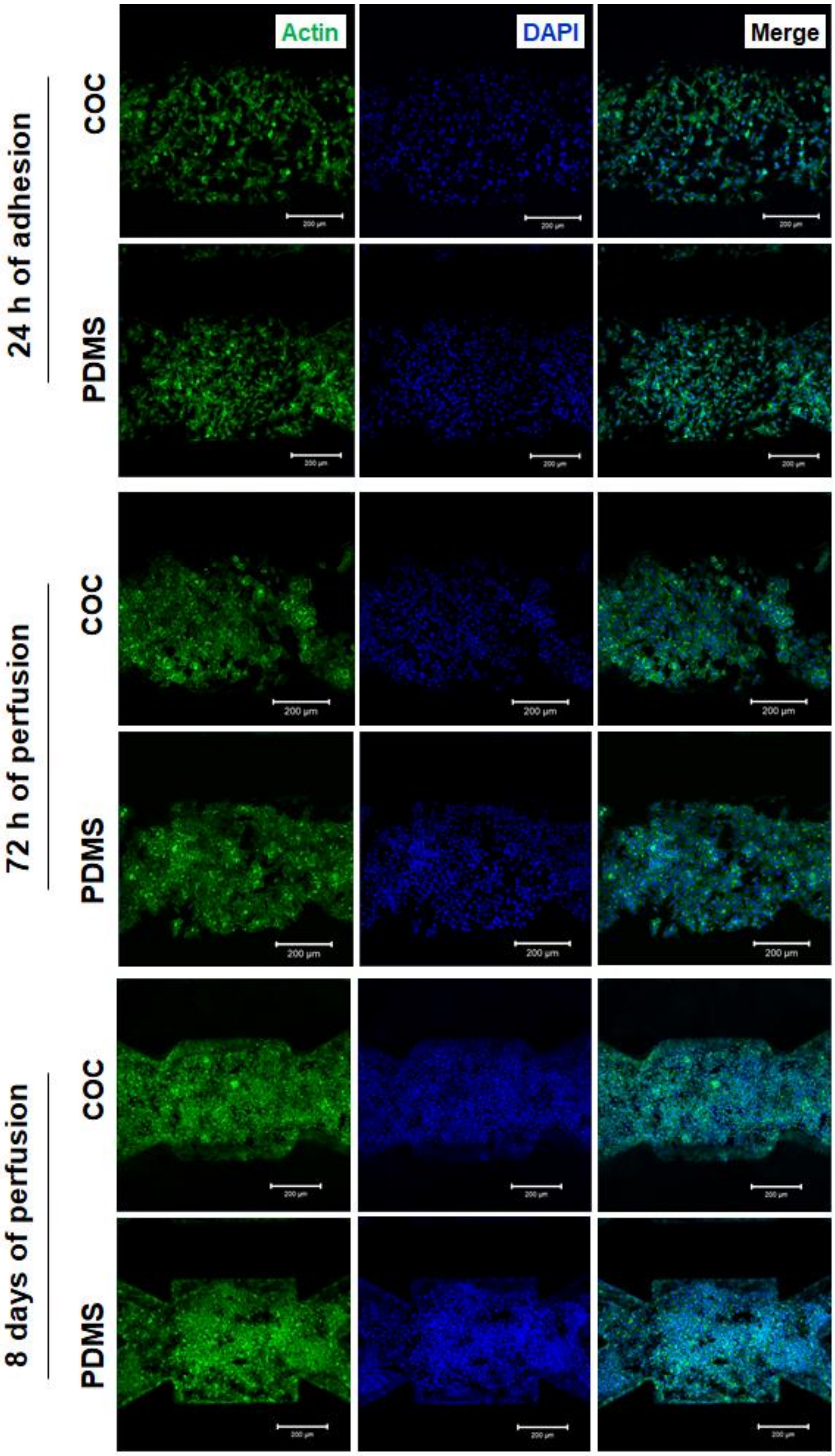

Figure S3. Phalloidin and DAPI stainings of HepG2/C3a cells at different steps of experimen: DAPI (nuclei, blue) and phalloidin (F-actin, green); scale bar 200 µm.
